# Drink driving engagement in women: An exploration of context, hazardous alcohol use, and behaviour

**DOI:** 10.1371/journal.pone.0222195

**Published:** 2019-09-10

**Authors:** Kerry A. Armstrong, James E. Freeman, Jeremy D. Davey, Rachel L. Kelly

**Affiliations:** 1 Road Safety Research Collaboration, University of the Sunshine Coast, Sippy Downs, Queensland, Australia; 2 Centre for Accident Research and Road Safety–Queensland (CARRS-Q), Queensland University of Technology (QUT), Kelvin Grove, Queensland, Australia; Tongii University, CHINA

## Abstract

**Background:**

While drink driving continues to be significantly more common among male drivers, there is evidence from many countries that shows a growing trend of women engaging in this risky behaviour. The aims of the current study were threefold: (i) determine to what extent a sample of women drivers reported engaging in drink driving behaviour by expanding the construct into a range of definitions, (ii) determine if there were significant differences in self-reported engagement in drink driving behaviours in accordance with hazardous drinking behaviour, and (iii) identify which situational or personal factors would increase women drivers’ likelihood to engage in drink driving through presenting a range of scenarios.

**Method:**

Data were collected using an on-line, purpose-designed survey and promoted to reach women aged 17 years and older, living in Queensland, Australia. In addition to questions relating to demographic characteristics, participants completed items relating to engagement in seven drink driving related behaviours in the previous 12-month period, hazardous drinking as measured by the Alcohol Use Disorders Identification Test, and likelihood of driving when unsure if over the legal limit for licence type across a range of scenarios manipulating different situational factors. A total of 644 valid responses were received in the two-week period the study was advertised.

**Results:**

The results demonstrate women’s self-reported engagement in drink driving behaviour ranged from 12.6% (driving when they believed they were over the legal limit) to over 50.0% (driving when unsure if over the legal limit the morning after drinking alcohol) and was significantly more likely among those who reported hazardous levels of alcohol use. Circumstances in which women reported they would drive when unsure if over the legal BAC limit were when they were a few blocks from home, if they subjectively felt they were not too intoxicated, or if they needed their car to get somewhere the next morning.

**Conclusion:**

Examining drink driving behaviour by way of responses to nuanced definitions provided valuable insight into self-reported engagement in the behaviour and highlights the usefulness of multi-measure dependent variables in order to illuminate a more accurate acknowledgement into both the type (and extent) of drink driving behaviours.

## Introduction

Drink driving is a major road safety concern worldwide and remains a major contributor of road injuries and fatalities in Australia, accounting for over 10% of road fatalities each year [1]. This is despite the significant efforts undertaken to address the issue in Australia including legislative Blood or Breath Alcohol Concentration (BAC) limits, random breath testing, and mass media campaigns to deter road users from engaging the behaviour to legal sanctions and education and rehabilitations programs for those that have been apprehended.

Drinking culture is an important part of Australia’s national identity [2–5]. Cultural acceptance of alcohol consumption in Australia can be attributed to factors such as its central role in individual, family, and national rituals and celebrations [4]. While the use of alcohol in Australia is historically and closely tied to bonding and solidarity for men, broader cultural changes in Australia such as the changing roles of women have important implications for the culture of drinking among women [4]. A national poll of 1,820 Australians aged 18 years and over conducted by the Foundation for Alcohol and Research Education [6] reported that 78% of participants were concerned about the consequences of excessive drinking had on road traffic incidents.

Recent crash statistics report 8,197 drink driving related crashes in Queensland between 1 January 2008 to 31 December 2017 (written communication, Department of Transport and Main Roads, 5 December 2018). Approximately 20.0% of the alcohol related crashes in Queensland involved female drivers and riders. During the same period, 3,228 women were injured in a drink driving crash, of which 45.2% involved a female drink driver/rider. Of all drink driving apprehensions across Queensland in the period 1 January 2008 to 31 December 2017 (N = 248,522), women comprised 20.9% (n = 52,028; written communication, Department of Transport and Main Roads, 5 December 2018). Current Australian drink driving law stipulates it is an offence for any road user to operate a vehicle with a BAC equal to or greater than 0.05, commonly referred to as the general alcohol limit. In most jurisdictions, a BAC of zero applies to learner drivers, those who hold a provisional licence (P1 or P2) and professional or heavy vehicle drivers. This is generally referred to as the ‘no alcohol’ limit. Queensland has in place a Graduated Licensing System that takes a stepped approach and permits newly licence drivers to progress (with restrictions) through to an open (i.e., unrestricted) driver’s licence. The program consists of a learner period where driving is supervised, a two-stage provisional period where a number of restrictions are placed on driving (e.g., night travel and passengers; use of hand-held and hands-free mobile phone technology; high-powered vehicle restrictions). It is during the learner and provisional stages that the ‘no alcohol’ or zero BAC limit is required when driving, regardless of age. Once drivers attain an open licence (a minimum of four years from the commencement of the learner licence phase), the general alcohol limit is increased to less than 0.05.

Overall, drink driving is significantly more common among male drivers; however, recent research shows a growing trend of women engaging in in drink driving in multiple countries across the world including Australia, New Zealand, Canada, the United States, United Kingdom, Finland, Sweden, and Germany [7–11]. Slade and colleagues reviewed 68 drink driving studies, with the majority of studies conducted in Europe (39.7%) and North America (36.7%) [10]. These studies collected data from 1948 to 2014, and data were analysed stratified by five-year birth cohorts from 1891 to 2001. Male-to-female ratios, representing the relationship between male and female levels of alcohol use and related harms, were examined. Results showed a closing gender gap in general (any) alcohol use, problematic alcohol use, and alcohol-related harm (including drink driving incidents). This closing gender gap is linked to the rising trend in alcohol consumption and detection of drink driving among women and is sometimes accompanied by a reduction or stabilisation in rates of drink driving among men [10, 12].

These findings suggest drink driving is an increasing problem for women, which highlights the need for gender-specific research and strategies to reduce alcohol-related harm and associated drink driving behaviour. However, there remains very limited research regarding women who engage in drink driving behaviour and the factors that influence their decision to engage in this risky behaviour. Further, it is important that research relating to women’s drink driving behaviour recognises the unique sex-related (biological) and gender-related (psycho-socio-cultural) vulnerabilities they have to alcohol compared to men. For instance, women display a higher BAC compared to men after consuming the same amount of alcohol [13, 14], have a greater vulnerability to the effects of binge drinking [15], take longer to recover from the cognitive impairments associated with alcohol consumption [16], and require more time to return to a zero BAC [17]. These vulnerabilities coalesce in women displaying a greater degree of impairment in driving simulations and in cognitive-behavioural assessments after consuming the same amount of alcohol/kg as men [18]. The difference in performance indicates that, through a range of bio-psycho-social-cultural factors, the women who engage in drink driving are at greater risk, however research in this area has gone largely unacknowledged as men report engaging in drink driving more frequently.

A foundational aim of the current study was to address the gaps outlined in the literature by determining to what extent a sample of women drivers reported engaging in drink driving behaviours. Further, the current study is the first (to the authors’ knowledge) to undertake a wider exploration into the frequency of drink driving events by expanding the construct into a range of different components. The reason for such an undertaking is multi-fold, including a recognition that: (i) a drink driving episode can be manifested in a range of different ways stemming from deliberate violations (e.g., above the legal BAC for licence type) to an event containing some level of uncertainty about whether the driver was (in fact) over the legal limit at the time of driving a vehicle (e.g., following morning after heavy drinking), (ii) research has repeatedly demonstrated the inaccurate nature of human memory [19], and thus a broader exploration was undertaken to operationalise the variable of interest and (iii) a variety of self-report biases have been suggested to negatively impact upon collected self-report data [20], and therefore, ‘broader’ definitions of drink driving (e.g., instances of ambiguity) were included in the corresponding analysis in order to capture (and analyse) possible drink driving events. For example, and at the very least, events of motor vehicle operation where the driver is “unsure” if they were over the legal limit can clearly be considered a high-risk situation that should be included in complementary analysis into strategies that mitigate such risk. More broadly (and from a different methodological perspective), it is noteworthy that self-reported deterrence-based research (focusing on a range of legal and non-legal sanctions) has generally failed to capture a high level of variance in regression analyses when aiming to predict offending behaviours e.g., >30% [21, 22]. It may be suggested that this limitation is (in part) due to research commonly relying on single-item dependent variables (rather than multiple items), which can naturally reduce psychometric robustness such as construct validity. In regard to the latter, the authors are unaware of any previous attempts to examine the construct of drink driving from a multi-dimensional perspective. But rather, published research continues to focus heavily on the measurement of a single-item as the dependent variable. Given this, it is argued that there is a need to conduct exploratory research into the usefulness of innovative multi-measure dependent variables in order to illuminate a more accurate acknowledgement into both the type (and extent) of drink driving behaviours. Currently, this issue has been completely overlooked in the drink driving research domain and the current study will attempt to address this gap.

A secondary aim was to determine if there were significant differences in self-reported engagement in drink driving behaviours in accordance with hazardous drinking behaviour. In a recent study by Bender and colleagues showed that women with multiple drink driving offences were more likely than first-time offenders to have more severe alcohol-use disorder symptoms [23]. The authors suggested that alcohol-use disorder severity is the main factor contributing to recidivism among women. However, it is important to note that the study involved a relatively small sample with a likely bias towards women with more severe alcohol use disorder, and a cross-sectional (rather than longitudinal) design. Given this, the current study will use the Alcohol Use Disorders Identification Test (AUDIT) to determine if women who engage in hazardous drinking are more likely to engage in drink driving behaviour compared to low-risk drinkers.

The final aim of the current study was to identify which situational or personal factors would increase women drivers’ likelihood to engage in drink driving through presenting a range of scenarios. Attribution theory explains that a number of factors, including circumstances and consequences, impact on a person’s perception regarding the risks associated with drink driving. For example, people are often more willing to engage in drink driving behaviour when they only need to go a short distance or when they are familiar with the roads [24–26]. However, additional factors may affect women when deciding to drive after consuming alcohol. A qualitative study by Robertson and Ireland [12] reported that personal safety after attending social events is noted as a key concern and related to both a lack of alternative transportation (e.g., buses, taxis) and risk to safety while waiting for public transport late at night. Some women also expressed safety concerns associated with staying overnight at the location where they had been drinking alcohol (e.g., when attending a house party). Within these scenarios, the risks for personal safety could be considered equal to risks for drink driving, impacting on women’s decision to drive after drinking alcohol.

## Method

### Participants

Participants comprised 644 women from across Queensland, Australia, aged between 17 and 67 years. Participants were recruited using a paid Facebook advertisement that was promoted for two-weeks during the Queensland school holiday period, September to October 2018. The mean participant age was 29.22 years (*SD* = 11.22).

### Measures

The dependent variables operationalised for use in the purpose-designed survey were developed as a result of previous qualitative work undertaken by the first three authors. The survey was designed to capture the nuances of women’s engagement in drink driving, using the independent variable of licence type was used to address the dependant variables (drink driving behaviour). In addition, demographic information was obtained from participants that detailed their age, category of licence held, hours spent driving each week (exposure), marital status, education level, employment status, household income, and if they had, in the previous 12-month period, changed their route in order to avoid the possibility of being stopped for a roadside breath test.

#### Definitions of drink driving behaviour

The variables operationalised for use in the current study focused on the frequency of engagement in seven drink driving related behaviours in the previous 12-month period. Participants were asked to respond to each of the questions (e.g., How often have you driven after drinking any amount of alcohol in the past 12 months?) using a 7-point Likert scale ranging from 1 ‘never’ to 7 ‘very often’.

#### Hazardous drinking

Hazardous drinking was measured by the Alcohol Use Disorders Identification Test (AUDIT), an alcohol screening measure developed by the World Health Organization (WHO) to identify harmful and risky drinking [27]. The AUDIT consists of 10 items which are scored on a 0 to 4 ordinal scale (yielding a range of 0–40) where higher scores indicates a higher probability of harmful/hazardous drinking. The use of an overall score to indicate levels of harmful/hazardous drinking is most commonly applied in the literature. The scale authors recommended a cut-off score of 0–7 for low-risk drinking or abstinence, a cut-off of 8–15 scores for moderate risky drinking, 16–19 for high-risk drinking, and 20–40 for possible dependence [27]. Studies have found that the AUDIT has a lower sensitivity for women than for men at the cut-off score of 8 (27% lower) and 7 (24% lower), and a review of studies using the AUDIT have found additional support for lowering the cut-off for women to 5 or 6 [28]. However, the current study used an AUDIT cut-off score of equal to and greater than 8 to identify hazardous drinkers.

#### Drink driving scenarios

Participants were presented with a range of scenarios manipulating different situational factors and asked how likely they were to drive when unsure if they were over the legal limit for their licence type. Examples include: ‘Home is close by (i.e., just a few blocks away)’, and ‘If needing to get somewhere the following morning after a night of drinking’, with ten scenarios presented in total. Responses were recorded using a 7-point Likert scale ranging from 1 ‘very unlikely’ to 7 ‘very likely’.

### Procedure

Following ethical approval from the Queensland University of Technology, Human Research Ethics Committee, a paid Facebook advertisement was developed and targeted to reach women aged 17 years and older, living in Queensland, Australia. Those who saw the advertisement and interested were directed to the study page for further information, with 1,454 page visits during the promotion period. To meet the selection criteria, potential participants were informed they were required to hold a valid Queensland licence, operate a vehicle for at least one hour per week, and have drunk alcohol within the preceding 12-month period. Those interested in taking part in the study were directed to the purpose-designed online survey by selecting the hyperlink provided on the study page. Consent was obtained by way of the participant selecting a hyperlink in which they agreed they had read the on-line participant information sheet and agreed to take part in the survey. This hyperlink took potential participants to the online survey itself. If the potential participant selected the hyperlink stating they had not read the participant information sheet and/or they did not consent to participant in the survey, they were taken to an end page where they were thanked for their interest. Those aged 17 years were not required to obtain consent from parents or guardians as their participation (as was the case for persons who participated) was entirely voluntary, their responses were not linked to them in anyway, were not obligated to respond to any questions they did not want to respond to, and they could withdraw at any time before submitting their survey responses without comment or penalty. Further, potential participants were required to respond to the screening questions before commencing the survey itself. Anyone that did not provide a correct response was automatically directed to an end page thanking them for their time and reiterating the study was seeking responses only from a select cohort of participants. The online survey took approximately 20 minutes to complete and those who participated were invited to go into the draw to win one of 100, $20 shopping gift cards. In total, 758 potential participants commenced the online survey, with 644 participants completing enough questions to form a valid response and be included in the analysis.

## Results

All analyses were conducted using IBM Statistical Package for the Social Sciences (SPSS) Version 25. A total of 758 responses were recorded. Of those, 114 responses were excluded as the participants did not provide answers to the dependent variable questions or complete the survey. Therefore, a final number of 644 responses were included in the following analyses and the demographic characteristics of the sample are displayed in Table 1. The majority of women reported they were in a relationship, had finished school or a tertiary bachelor’s degree, were employed, and reported a total annual household income of $80,000 AUD or less. A total of 436 participants responded to the AUDIT, with mean scores showing normal alcohol use in this population; however, over a quarter of the sample had a score of 8 or more, indicating hazardous alcohol use. Hazardous alcohol use was more common among P1 and P2 licence holders than open licence holders. Finally, when asked whether participants changed their route home to avoid the possibility of being breath tested by police, 10.4% (*n* = 48 of a total 463 responses to the question) stated they had done so within the previous 12-month period. Of these 48 participants, 37.5% reported doing so once, 29.2% reported doing so twice, and 33.4% reported doing so three or more times (up to 10 times). This item is a good indicator of drink driving engagement as only individuals who believed they could be drink driving would alter their behaviour to avoid apprehension.

**Table 1:** Socio-demographic characteristics of female participants by licence type.

	P1 licence n = 50 (7.8%)	P2 licence n = 117 (18.2%)	Open licence n = 477 (74.1%)	Total N = 644
Marital Status				
Single	28 (56.0%)	61 (52.1%)	118 (24.8%)	207 (32.2%)
In a relationship	22 (44.0%)	56 (47.9%)	334 (71.2%)	412 (64.1%)
Divorced or separated	-	-	19 (4.0%)	19 (3.0%)
Widow	-	-	5 (1.1%)	5 (0.8%)
Education				
High school (Years 10 or 12)	39 (78.0%)	85 (72.7%)	119 (25.0%)	243 (37.7%)
Trade certificate/apprenticeship	5 (10.0%)	6 (5.1%)	58 (12.2%)	69 (10.7%)
Bachelor / postgraduate degree	5 (10.0%)	19 (16.2%)	271 (56.8%)	295 (45.9%)
Other	1 (2.0%)	7 (6.0%)	29 (6.1%)	37 (5.7%)
Employment				
Full-time/part-time /casual	17 (34.0%)	48 (41.0%)	320 (64.4%)	372 (57.8%)
Unemployed/retired	3 (6.0%)	-	36 (7.5%)	39 (6.1%)
Student	11 (22.0%)	32 (27.3%)	55 (11.5%)	98 (15.2%)
Student and employed	19 (38.0%)	37 (31.7%)	54 (11.3%)	110 (17.1%)
Paid leave	-	-	25 (5.3%)	25 (3.9%)
Annual Household Income				
$0 to $40,000	27 (56.3%)	50 (43.9%)	101 (21.4%)	178 (28.0%)
$40,001 to $80,000	5 (10.4%)	25 (21.9%)	136 (28.8%)	166 (26.1%)
$80,001 to $120,000	4 (8.3%)	22 (19.3%)	106 (22.3%)	132 (20.8%)
$120,001 to $160,000	6 (12.5%)	8 (7.0%)	63 (13.3%)	77 (12.1%)
$160,001 to $200,000	4 (8.3%)	4 (3.5%)	42 (8.9%)	50 (7.9%)
$201,000 +	2 (4.2%)	5 (4.4%)	25 (5.2%)	32 (5.0%)
Average Driving Hours per week	8.74 (7.36)	8.21 (6.24)	9.17 (6.73)	8.96 (6.70)
AUDIT				
Total scale score: Mean (SD)	6.75 (4.59)	6.00 (4.78)	5.07 (4.36)	5.35 (4.48)
Score of 8 or greater: n (%)	10 (35.8%)	26 (32.1%)	76 (23.2%)	112 (25.7%)

### Drink driving behaviours

Results of the descriptive analysis of the various drink driving (DD) definitions are displayed in Table 2. One-way ANOVAs were conducted for each definition to determine if there were any significant differences between licence types. Levene’s test of homogeneity was breached for DD1, DD2, DD3, DD4, DD6, and DD7. This indicates a difference in variance between the licence groups, which makes sense in the context of the difference in legal BAC limits for provisional licence holders compared to open licence holders. Therefore, those with an open licence can report with more variability than those with a provisional licence. The ANOVA for DD5 revealed a non-significant Levene’s *F* statistic so can be reported as normal. For the other DDs, the Welch and Brown-Forsythe adjusted ANOVAs were conducted and, where congruent, the Welch statistic has been reported as it is the more conservative test.

**Table 2:** Descriptive results of women participants by licence type and drink driving definition.

Dependent variables	Total (*N* = 644)	P1 (*n* = 50)	P2 (*n* = 117)	Open (*n* = 477)
	Mean (SD)	Mean (SD)	Mean (SD)	Mean (SD)
DD1: How often have you driven after drinking any amount of alcohol in the past 12 months	2.53 (1.65)	1.42 (0.84)^a^	1.50 (1.01)^a^	2.90 (1.68)^b^
DD2: How often have you driven after drinking alcohol but confident you were under the legal limit for your licence type in the past 12 months	2.67 (1.86)	1.38 (0.81)^a^	1.49 (1.08)^a^	3.10 (1.90)^b^
DD3: How often have you driven within an hour of drinking two or more standard drinks in the past 12 months	1.54 (1.18)	1.10 (0.46)^a^	1.14 (0.52)^a^	1.69 (1.31)^b^
DD4: How often have you driven after drinking alcohol and were unsure if you would be over the legal limit for your licence type in the past 12 months	1.47 (1.04)	1.38 (0.88)^a^	1.27 (0.72)^a^	1.53 (1.11)^a^
DD5: How often have you driven when you believed you were over the legal limit for your licence type in the past 12 months	1.23 (0.75)	1.20 (0.67)^a^	1.17 (0.61)^a^	1.25 (0.78)^a^
DD6: How often have you driven the morning after drinking alcohol and were unsure if you were over the legal limit for your licence type in the past 12 months	2.10 (1.45)	2.16 (1.39)^ab^	2.60 (1.61)^a^	1.97 (1.39)^b^
DD7: How often have you driven the morning after drinking alcohol and believed you were over the legal limit for your licence type in the past 12 months	1.45 (1.06)	1.48 (0.97)^a^	1.69 (1.30)^a^	1.38 (1.00)^a^

*Note*: Significant differences indicated by superscript letters across the licence groups.

For DD1, the total average indicates that at some point in the prior 12 months, the women sampled did drive after drinking *some* amount of alcohol. By dichotomising the Likert responses with 1 representing never engaging in the behaviour and 2–7 representing some engagement, 64.3% (*n* = 414) of participants reported driving after drinking some amount of alcohol in the previous 12-month period. When isolated to licence type, the results logically indicated that open licence holders reported that they had driven after drinking any amount of alcohol more frequently than P1 or P2 licence holders in the previous 12 months. However, of interest is the higher mean reported by women with a P2 licence over those with a P1 licence, despite the zero BAC limit applying to both. The Welch adjusted ANOVA revealed that some of these licence differences were significant (*F*(2, 152) = 90.44, *p* < .001). Post hoc analyses using the Scheffé criterion for significance indicated that women with an open licence drove after drinking alcohol significantly more frequently than those with a P1 (*SE* = 0.23, *p* < .001) or P2 (*SE* = 0.16, *p* < .001) licence in the prior 12 months. There was no significant difference between those with a P1 or P2 licence (*SE* = 0.26, *p* = .948).

A similar pattern emerged for DD2 with how frequently the women drove after drinking alcohol but were *confident* they were under the legal limit for their licence type in the past 12 months. Using the dichotomised responses of 1 representing never engaging in the behaviour and 2–7 representing some engagement, 62.4% (*n* = 402) of participants reported driving after drinking some amount of alcohol in the previous 12-month period when confident they were under the legal limit for their licence type. The Welch adjusted ANOVA reported there was some significant differences between licence types (*F*(2, 165) = 103.78, *p* < .001). Scheffé-adjusted post hoc comparisons indicated that women with an open licence reported engaging in the behaviour more frequently than those with a P1 (*SE* = 0.26, *p* < .001) and P2 (*SE* = 0.18, *p* < .001) licence within the prior 12-months. There was no significant difference between those with a P1 or P2 licence (*SE* = 0.29, *p* = .934).

DD3 proposed a scenario of driving within one hour of drinking two or more standard drinks. This scenario would result in a BAC that was close to the 0.05 BAC limit for open licence holders and over the zero BAC limit for provisional licence holders. The total mean is much lower for this scenario than the previous two, with the majority of participants reporting they had never driven within one hour of consuming two or more standard drinks in the past 12-months. Nevertheless, having a mean greater than 1 implies that some participants engaged in this behaviour at least once in the previous 12 months. Dichotomising responses using the same method outlined above revealed 24.5% (*n* = 158) of participants fell into the latter category. The Welch adjusted ANOVA was significant (*F*(2, 176) = 31.33, *p* < .001). Scheffé-adjusted post hoc comparisons indicated that open licence holders drove within an hour of consuming two or more standard drinks more frequently than P1 (*SE* = 0.17, *p* = .003) and P2 (*SE* = 0.12, *p* < .001) licence within the prior 12 months. There was no significant difference between those with a P1 or P2 licence (*SE* = 0.20, *p* = .982).

In comparison to the second drink driving definition, DD4 assessed how frequently women drove while unsure if they were over the legal limit for their licence type in the previous 12-month period, and DD5 assessed how frequently women drove believing they were over the legal limit for their licence type. On average, majority of women reported never driving while being unsure or knowing they were over the legal limit. However, the dichotomised responses revealed a relatively large portion of women reported they drove despite being uncertain (*n* = 152; 23.6%) or when they believed they were (*n* = 81; 12.6%) over the legal limit for their licence type at least once during the prior 12-month period. The Welch adjusted ANOVA for DD4 was significant (*F*(2, 128) = 4.56, *p* = .011). However, Scheffé-adjusted post hoc comparisons revealed no significant differences between licence types. Conversely, the ANOVA for DD5 was not significant (*F*(2, 641) = .592, *p* = .553). Taken together, these results suggest that despite the reported engagement in possible or actual drink driving events, there were no significant differences between P1, P2, and open licence holders.

Both DD6 and DD7 relate to driving the morning after a drinking alcohol within the previous 12-month period. The descriptive results from DD6 assessed how frequently women drove the morning after a night of drinking while being unsure if they were over the legal limit for their licence type, and DD7 assessed how frequently women drove the morning after a night of drinking when they believed they were over the legal limit. Again, when the responses were dichotomised, half of the sample of women reported they had driven the morning after drinking alcohol when they were unsure if they were over the legal limit (*n* = 322; 50.0%); whereas over one-fifth (n = 139; 21.6%) drove the following morning when they believed they were over the legal limit for their licence type at least once during the prior 12 months. The Welch adjusted ANOVA for DD6 was significant (*F*(2, 133) = 7.65, *p* = .001). Scheffé-adjusted post hoc comparisons indicated that P2 licence holders reported driving the next morning when they were unsure if they were over the legal limit more frequently than open licence holders (*SE* = 0.15, *p* < .001). However, there was no significant difference between P1 and P2 licence holders (*SE* = 0.24, *p* = .196), or between P1 and open licence holders (*SE* = 0.21, *p* = .662). Finally, while the Brown-Forsythe adjusted ANOVA for DD7 was significant (*F*(2, 196) = 3.59, *p* = .029), the Welch adjusted ANOVA was not (*F*(2, 112) = 2.95, *p* = .056). Given the Welch adjusted ANOVA is a more conservative test, the decision was made to accept the non-significant result.

### Hazardous alcohol use

To determine if there was a relationship between hazardous alcohol use and participants’ responses to five of the seven DDs (DD3 through to DD7), bivariate correlation analysis was conducted. The correlation coefficient between DD1 and DD2 was .942, *p* = < .0001; revealing multicollinearity. It was interpreted both questions were measuring the meaning of DD2 (i.e., “driving when confident of being under the legal BAC limit”), which is not reflective of engagement in drink driving behaviour and therefore was not included in the subsequent.

The responses for each of the five DD’s were dichotomised into two groups, with those who reported they had never engaged in the drink driving behaviour (i.e., response of 1 on the 7 point Likert scale) and those who responded they had engaged in the drink driving behaviour (responses ranging from 2 through to 7 on the Likert scale; indicating they had engaged in the behaviour at least once in the past 12 months). The two groups are referred to as non-drink drivers (NDDr) and drink drivers (DDr) respectively.

Total AUDIT scores were also used to create two groups, with the referent group categorised as those who scored 8 or greater and therefore categorised as hazardous drinkers. The dichotomised AUDIT results were then correlated with each of the five DDs, the results of which are displayed in Table 3. Chi square tests of independence were also conducted to determine if there were significant differences between hazardous alcohol use in dichotomised drink driver and non-drink driver groups (see Table 3).

**Table 3:** Correlation coefficients and chi-square analysis between dichotomised AUDIT scores and varying definitions of drink driving behaviour.

Drink Driving Definitions	AUDIT Score Correlation Coefficient (referent group—hazardous alcohol use)	Drink Drivers (DDr) and Non-Drink Drivers (NDDr) Categories	Hazardous Alcohol Use on AUDIT		Significance Level
			Total AUDIT Score ≤ 7	Total AUDIT Score ≥ 8	
DD3: How often have you driven within an hour of drinking two or more standard drinks in the past 12 months	.25**	DDr	18.2%	41.1%	χ^2^ (df1) = 23.793, *p* = < .001
		NDDr	81.8%	58.9%	
DD4: How often have you driven after drinking alcohol and were unsure if you would be over the legal limit for your licence type in the past 12 months	.34**	DDr	16.0%	47.3%	χ^2^ (df1) = 44.519, *p* = < .001
		NDDr	84.0%	52.7%	
DD5: How often have you driven when you believed you were over the legal limit for your licence type in the past 12 months	.34**	DDr	5.2%	30.4%	χ^2^ (df1) = 50.807, *p* = < .001
		NDDr	94.8%	69.6%	
DD6: How often have you driven the morning after drinking alcohol and were unsure if you were over the legal limit for your licence type in the past 12 months	.51**	DDr	35.5%	83.9%	χ^2^ (df1) = 78.233, *p* = < .001
		NDDr	64.5%	16.1%	
DD7: How often have you driven the morning after drinking alcohol and believed you were over the legal limit for your licence type in the past 12 months	.46**	DDr	9.6%	51.8%	χ^2^ (df1) = 91.311, *p* = < .001
		NDDr	90.4%	48.2%	

Note

* *p* < .05

** *p* < .0001.

The AUDIT responses were significant and positively correlated with all of the DDs, revealing that women who reported hazardous alcohol use also reported higher frequency of engaging in drink driving behaviour in the past 12 months. The strongest relationship was observed for DD 6 and DD 7, which revealed an association between hazardous alcohol use and engagement in drink driving behaviour the morning after a night of drinking alcohol. Further, the correlation coefficient was the same for DDs 4 and 5, revealing the degree of association between hazardous alcohol use and engagement in possible (i.e., when you were unsure if over the legal BAC limit) as well as probable (i.e., when you believed you were over the legal BAC limit) drink driving was equal.

Significant differences were observed across all chi square tests, revealing differences between alcohol use (i.e., hazardous alcohol use compared to non-hazardous alcohol use) and the drink driving DDs. For all DDs, women who reported engaging in hazardous alcohol use were also more likely to engage in drink driving behaviour typified by each DD when compared to women who reported non-hazardous alcohol use. Similarly, women who reported non-hazardous alcohol use were more likely to report never engaging in drink driving behaviour typified by each DD when compared to women who reported hazardous alcohol use. Of interest, over 80% of women who reported engaging in hazardous alcohol use reported they drove the morning after drinking alcohol when they were unsure if they were over the legal limit for their licence type. This proportion fell to just over 50% for driving the morning after while believing they were over the limit. These results demonstrate that women who engage in hazardous alcohol use are seeing the greatest impact on their driving the following morning after an evening of drinking alcohol.

### Drink driving scenarios

A between-groups ANOVA was conducted to determine if women would drive when they were unsure if they were over the legal limit for their licence type across a number of scenarios. Table 4 displays participants’ likelihood of engaging in possible drink driving behaviour according to licence type. Overall, the means across all scenarios and licence types were low, which indicates that most women believed they were unlikely to drive when they were unsure if they were over the legal limit for their licence type regardless of the scenario proposed. However, participants were more likely to report engaging in some scenarios compared to others. For example, regardless of licence type, women were more likely to drive when they were uncertain if they were over the legal limit for their licence type if they were close to their place of residence (e.g., a few blocks from home), if they subjectively felt they were ok to drive (e.g., not too intoxicated), or if they needed their car to get somewhere the next morning.

**Table 4:** Means, standard deviations, and tests of significance between licence types and various scenarios where participants may drive while unsure if they were over the legal limit.

Scenario *Response Scale* *1*: *Very Unlikely–* *7*: *Very Likely*	Total	P1	P2	Open	*F statistic*
	Mean (*SD*)	Mean (*SD*)	Mean (*SD*)	Mean (*SD*)	
1: If home is close by (i.e., just a few blocks away)	2.77 (2.08)	2.36 (1.69)	2.69 (1.90)	2.83 (2.16)	1.61
2: If you feel perfectly ok to drive (i.e., don’t feel tipsy, wobbly, or drunk)	2.59 (1.94)	1.91 (1.25)	2.43 (1.89)	2.70 (2.00)	3.18*
3: If needing to get somewhere the following morning after a night of drinking	2.38 (1.98)	1.93 (1.41)	2.56 (2.00)	2.38 (2.02)	.20
4: If you don’t want to leave your car behind	2.08 (1.73)	1.53 (1.04)	2.03 (1.62)	2.14 (1.81)	7.96**
5: If it was only a short drive home (only 10–15 mins)	2.06 (1.68)	1.71 (1.14)	1.88 (1.38)	2.14 (1.78)	1.85
6: If travelling by yourself would feel unsafe	2.01 (1.67)	1.87 (1.27)	1.91 (1.56)	2.05 (1.74)	7.03**
7: If using public transport would be inconvenient	1.98 (1.73)	1.53 (0.94)	1.64 (1.32)	2.10 (1.87)	.48
8: If taking an Uber or taxi would be expensive	1.95 (1.71)	1.71 (1.27)	1.76 (1.55)	2.02 (1.78)	5.93**
9: If you’re not expecting to see police/RBT on the route home	1.91 (1.60)	1.62 (1.11)	1.90 (1.60)	1.95 (1.64)	.85
10: If feeling pressured by friends to drive	1.34 (0.90)	1.31 (0.82)	1.30 (0.82)	1.35 (0.93)	2.65

Note

* *p* < .05

** p < .01

*** p < .001.

When analysed by licence type, between groups ANOVA results showed that P1 licence holders were the least likely cohort to report engaging in any of the scenarios if they were unsure if they were over the zero BAC limit. Open licence holders, on the other hand, reported they were more likely than P1 or P2 licence holders to drive when they were unsure if they were over the general BAC limit of 0.05 in all but one scenario. For that particular scenario, P2 licence holders were more likely to drive when unsure if they were over the zero BAC limit if they needed to get somewhere the following morning after a night of drinking.

A number of between groups ANOVAs were conducted to determine if there were any significant differences by licence type. The unequal variances between licence type resulted in significant Levene’s test of homogeneity for scenario 1, scenario 2, scenario 4, scenario 5, scenario 6, scenario 8, and scenario 10. Therefore, the Welch adjusted ANOVA was used as a more conservative test. Levene’s *F* statistic was not significant for scenario 3, scenario 7, or scenario 9 so the ANOVA for those corresponding scenarios is reported as normal. ANOVA *F* statistics are also displayed in Table 4 (over page), which indicate significant differences in likelihood of engaging in a number of scenarios when unsure if over the legal limit for their licence type.

Significant differences were observed between P1, P2 and open licence holders for scenario 2, scenario 4, scenario 6, and scenario 8. Scheffé-adjusted post hoc comparisons were conducted with the scenarios with significant main effects to investigate differences by licence type. Despite a significant ANOVA, the post-hoc comparisons for scenario 2 revealed no significant differences between licence types. For scenario 4, comparisons showed that open licence holders were significantly more likely to drive when unsure if over the legal limit than P2 licence holders when they did not want to leave their vehicle behind (*SE* = 0.19, *p* = .043). There were no significant differences between P1 and P2 licence holders (*SE* = 0.31, *p* = .942) or between P1 and open licence holders (*SE* = 0.27, *p* = .107). For scenario 6, comparisons showed that open licence holders were significantly more likely to drive when unsure if over the legal limit than P1 licence holders when they felt unsafe to travel home by themselves (*SE* = 0.31, *p* = .037). There were no significant differences between P1 and P2 licence holders (*SE* = 0.35, *p* = .326) or between P2 and open licence holders (*SE* = 0.21, *p* = .429). Finally, despite the significant ANOVA result for scenario 8, there were no significant differences between the likelihood of whether each licence type would drive when unsure if over the legal limit when taking an Uber/taxi would be too expensive.

As descriptive results consistently showed, open licence holders reported they would be more likely to drive when unsure if they were over the legal limit for their licence type in all but one scenario. As such, it can be seen that experience does not translate to safer driving behaviours. The scenarios described above reveal that women with the most driving experience demonstrated either more complacency or higher risk taking than their less experienced peers.

## Discussion

Current literature suggests increased engagement in drink driving among women in a number of countries across the world [7–11]. This growing engagement, combined with the unique sex-related bio-psycho-socio-cultural vulnerability to alcohol, highlighted the need for research that explores how and why women engage in drink driving. The current study is the first (to the authors’ knowledge) to undertake a wider exploration into the frequency of drink driving events by compartmentalising the construct into a range of definitions. Further, it expanded upon the understanding of drink driving behaviour among women by exploring the relationship between drink driving and hazardous alcohol use. Finally, a number of scenarios explored the circumstances in which women engage in the behaviour.

### Differences across definitions of drink driving behaviour

The dependent variables examined in the current study focused on the frequency with which participants engaged in a range of drink driving related behaviours in the previous 12-month period. Results showed that the most common behaviours participants reported were driving after drinking any amount of alcohol, driving after drinking alcohol while confident they remained under the legal BAC limit for their licence type, and driving the morning after a night of drinking alcohol but were unsure if they were over the legal limit. A noteworthy finding was the sizeable proportion of the sample that may have been at risk of engaging in a drink driving event. Driving after drinking any amount of alcohol was reported by all licence types including P1, P2, and open licence holders. While this behaviour is permissible for those with an open licence (providing they remain below the general alcohol limit of 0.05), P1 and P2 licence holders are restricted to a zero BAC limit.

This finding highlights possible engagement in drink driving behaviour(s) among young, newly licensed women. Although these newly licenced participants are not permitted to drive when they have any detectable amount of alcohol in their system, and should be cognisant of this restriction, a surprising high percentage of young (recently licensed) drivers are at risk of engaging in drink driving events. Young drivers who do drive after drinking alcohol are at a higher risk than more experienced drivers, as they are five times more likely to be involved in an alcohol-related crash at BAC levels lower than the legal limit (i.e. 0.02) [29]. In Queensland, Australia between 01 January 2008 to 31 December 2017, there were 8,197 crashes associated with drink driving of which 33.6% were among young drivers aged 16 to 24 years (written communication, Department of Transport and Main Roads, 5 December 2018). Despite being the focus of research and campaigns for over a decade, young drivers are still over-represented in terms of the number of crashes, injuries and deaths on Australian roads.

Examination of participant responses concerned with driving when “unsure if over the legal limit” and “when thought to be over the legal limit” revealed interesting results. For instance, a relatively large proportion of women reported they drove despite being uncertain (23.6%) or when they believed they were (12.6%) over the legal limit for their licence type at least once in the preceding 12-month period. This prevalence is in line with other studies examining self-report engagement in drink driving [30]. However, while Stephens and colleagues [30] defined self-report drink driving as when the participant believed they were over the limit, the current study demonstrates that the different definitions of drink driving produce different levels of self-report engagement. For example, a significant difference was observed between responses concerning if unsure or believing self to be over the limit, which provided important information regarding participants decision to drive in the 12 months prior. While women perceived this difference to be significant, Rossheim and colleagues [31] report that drivers often underestimate their BAC and this was particularly common among drivers 26 years and younger. Therefore, drivers who have consumed alcohol and are unsure if they are over the legal limit are still likely to be apprehended for drink driving.

When asked about driving the morning after drinking alcohol, half (50.0%) reported they had driven when they were unsure and 21.6% reported they had done so when they believed they were over the legal limit for their licence type. This is a dramatic increase from previously reported statistics, which showed that just under one fifth of participants reported they were possibly over the legal limit when they drove the morning after drinking alcohol [32]. As previously discussed, pharmacokinetic literature demonstrates that women take longer to metabolise alcohol than men, suggesting that women may be at greater risk of engaging in drink driving the morning following a night of drinking [17]. Through self-report data, the current study supports the literature and demonstrates that driving the morning after a night of drinking alcohol when unsure if over the legal BAC limit is commonly reported by women drivers in Queensland (in current times).

It is likely that the difference between being uncertain and believing yourself to be over the legal limit is based on the subjective assessment of feeling okay to drive and counting standard drinks as reported in the qualitative interviews. Both methods are highly unreliable as a plethora of individual factors (e.g., height, weight, health, medications, stress etc.) are known the influence BAC. Counting standard drinks is a rudimentary mechanism to avoid driving over the legal BAC limit, as it is impossible to account for all these factors; however, this strategy is culturally normalised among Australian female road users [33]. Given this, it is reasonable to interpret that some participants who reported being “unsure” were in fact culpable of driving over the legal limit. Research demonstrates that reaction time is impaired at a BAC as little as 0.02 and observed impairment in perception and psychomotor skills starts at a BAC of 0.04 [34].This is why there has been a focus on promoting the separation of alcohol use from driving across all Australian jurisdictions as well as the deliberate attempt to move away from counting the number of standard drinks to estimate BAC in road safety campaigns. However, it appears it is important to continue to reiterate this message and to ensure women are included as a focus in campaigns.

In sum, a significant finding of the current study is that the inclusion of various definitions for drink driving behaviour identify a higher number of women road users who have reported engaging in such behaviour. The results across all definitions of drink driving reveal that self-reported engagement in drink driving behaviour in the previous 12-month period among the current sample of Queensland women ranges between 12.6% (driving when believe over the legal limit) to over 50.0% (driving when unsure if over the legal limit the morning after drinking alcohol). The self-reported range is much greater than the apprehension rate of 20.9%, which is based on apprehensions rates for women across Queensland between 1 January 2008 and 31 December 2017 (written communication, Department of Transport and Main Roads, 5 December 2018).

### Differences between licence types

Examination of differences between women and licence type revealed some interesting results. Open licence holders reported greater engagement in the range of drink driving behaviours, supporting previous research that demonstrates that female drink drivers are typically older when compared to male drink drivers [9, 35]. However, those with a P2 licence reported the highest rate of driving the morning after drinking alcohol when they were unsure if they were over the legal limit as well as when they believed they were over the legal limit for their licence type. P1 licence holders also reported higher engagement in these two drink driving behaviours compared to open licence holders. One possible explanation for this finding could be that open licence holders believed the 0.05 BAC limit provided them with a buffer or margin of error, which in turn, gives them more confidence their BAC has reduced sufficiently overnight to be below the general alcohol limit by the following morning. An alternative explanation is that young persons are more likely to engage in heavy episodic drinking [36]. Women who engage in heavy episodic drinking (defined as more than four standard drinks within a single occasion) [37] would likely record a BAC greater than zero the following morning. As such, it is possible women with a provisional licence reported engaging in drink driving behaviour the following morning more so than open licence holders because of the amount of alcohol they consume, combined with the knowledge they are required to adhere to the zero BAC restriction.

### Hazardous drinking

A key interest of this paper was the relationship between AUDIT scores and the definitions of drink driving behaviour. Of the women sampled, 35.8% of P1, 32.1% of P2, and 23.2% of open licence holders were identified to engage in hazardous alcohol use. When scores on AUDIT were dichotomised into hazardous/non-hazardous alcohol use, it was found that hazardous alcohol use was positively correlated with each of the drink driving behaviours. Specifically, women who reported hazardous alcohol use were also most likely to drive the morning after drinking alcohol when they were unsure as well as when they believed they were over the legal limit for their licence type. This finding was confirmed when the dependent variables were dichotomised into drink drivers/non-drink drivers. Indeed, it was found that over 80% of women who reported engaging in hazardous alcohol use also reported they drove the morning after drinking alcohol when they were unsure if they were over the legal limit for their licence type. This proportion fell to just over 50% for driving the morning after while believing they were over the limit. While the majority of drink driving literature focuses on males, the results of the current study demonstrate that: (a) a sizable proportion of women drink hazardous amounts of alcohol and (b) women who engage in hazardous alcohol use are also at greatest risk of driving when over the legal BAC limit for their licence type the following morning. These results are consistent with other literature demonstrating hazardous alcohol use is associated with drink driving in both male and female drivers [30, 38] as well as older women [39]; and that heavy episodic drinking significantly increases engagement in drink driving behaviour [40].

### Scenarios for driving when unsure if over the legal BAC limit

Participants were presented with a range of scenarios manipulating different situational factors and asked how likely they were to drive when unsure if they were over the legal BAC limit for their licence type. Across all licence types, women were more likely to drive when uncertain if they were over the legal limit for their licence type if they were a few blocks from home, if they subjectively felt they were not too intoxicated, or if they needed their car to get somewhere the next morning. These findings support previous research that has found that drivers are more willing to justify driving when possibly over the legal BAC limit when they only need to travel a short distance or if they are familiar with the route [24–26]. While Robertson and Ireland [12] reported that women were more likely to drink drive when concerned about their safety, participants in the current study did not indicate they were more likely to drive when unsure if they were over the limit in this scenario than any of the other scenarios. These findings suggest that across a larger cross-sectional sample of women, factors relating to convenience emerged as more influential than safety when deciding to drive after consuming alcohol; however, further research is needed to clarify the contrasting results.

When analysed by licence type, open licence holders were shown to be significantly more likely than P1 and P2 licence holders to drive when uncertain if over the legal BAC limit when they did not want to leave their vehicle behind and when they felt unsafe travelling home by themselves. The implication of these findings is that driving experience does not always equate to safer driving behaviours. It may yet be found that some motorists become desensitised to either drink driving behaviours, or the risks associated with such behaviour. These scenarios demonstrate that women open licence holders are more comfortable with complacency or possible risk taking and supports previous literature demonstrating that low self-control and impulsivity were significantly associated with female drink drivers [41]. While not statistically significant, the only scenario where open licence holders did not report the highest average mean was when they needed to drive the morning after drinking alcohol. For this scenario, the highest mean was reported by P2 licence holders and adds further weight to the previously discussed findings, where provisional licence holders were more likely to drive the morning after drinking alcohol when possibly in excess of the zero BAC restriction.

### Implications for future research and policy

The current study adds to the literature around drink driving as it emphasises the value in including a range of definitions for drink driving behaviours. By doing so, it has revealed higher levels of engagement in some definitions of drink driving than in others. By highlighting the subtleties of women’s engagement of drink driving behaviours, future research can capture more nuanced information about the ways that people define and engage in drink driving behaviours. The results also provide support for the relationship between hazardous drinking and drink driving behaviours in a larger cross-sectional sample of women, as drink drivers across five different drink driving behaviours were significantly more likely to report hazardous levels of alcohol use than non-drink drivers. The scenarios explored in the current study demonstrate that women drivers consider convenience more so than safety when making the decision to drive when possibly over the legal limit. Further, older women drivers reporting higher levels of engagement in drink driving display a complacency or minimisation of risks relating to drink driving. Research has established that punishment avoidance, engaging in a behaviour without receiving a punishment, is the greatest predictor of future engagement in drink driving [42]. It will be of interest to researchers and policy makers whether women find current campaigns aimed at deterring drink driving as relevant to themselves. Future focus should look deeper into why women are increasingly engaging in drink driving [7–11], and at ways of increasing perception of deterrence through targeted campaigns that are relevant to women of all ages.

## Conclusion

In summary, through a cross-sectional sample of 644 Queensland women, the relative prevalence of women’s self-reported engagement in a comprehensive variety of drink driving behaviours, the relationship between drink driving engagement and self-reported hazardous alcohol use, and the scenarios in which women believed would engage in drink driving behaviours was explored. The results demonstrate that between 12.6% (driving when believed they were over the legal limit) to over 50.0% (driving when unsure if over the legal limit the morning after drinking alcohol) of women drivers’ report engaging in drink driving behaviour. Engagement in drink driving behaviours emerged as being significantly more likely among women who reported hazardous levels of alcohol use. Examination of the circumstances in which Queensland women engage in drink driving behaviour showed that across all licence types, women would drive when unsure if they were over the legal BAC limit if they were a few blocks from home, if they subjectively felt they were not too intoxicated, or if they needed their car to get somewhere the next morning. Continued research into the subtleties amongst self-reported engagement in drink driving will help inform future campaigns that deter drink driving among women of all ages.
